# Change in patent foramen ovale height is associated with cryptogenic stroke and the construction of a morphology-based scoring system

**DOI:** 10.3389/fcvm.2022.1010947

**Published:** 2022-11-28

**Authors:** Xiaoqin Liu, Yu Zhang, Hang Xie, Haowei Zeng, Jingyan Sun, Linjie Su, Bingyi Li, Xiaoyi Xue, Yushun Zhang

**Affiliations:** Department of Structural Heart Disease, First Affiliated Hospital, Xi'an Jiaotong University, Xi'an, Shaanxi, China

**Keywords:** cryptogenic stroke, patent foramen ovale, morphology, scoring system comparison, atrial septal aneurysm

## Abstract

**Introduction:**

Current guidelines recommended patent foramen ovale (PFO) occlusion as the preferred treatment for PFO-related cryptogenic stroke (CS); however, finding the causative foramen ovale remains challenging. This study aimed to identify predictors and establish a scoring system by assessing PFO morphology and stroke-related factors.

**Methods:**

Based on a prospective multicenter registered clinical trial, we compared data mainly derived from transesophageal echocardiography (TEE) and clinical history in patients with PFO-related CS and those without CS (non-CS) with incidental PFO. Subsequently, we explored independent predictors using logistic analysis, established a scoring system based on the results, and finally evaluated the scoring system using receiver operating characteristic (ROC) analysis and internal validation.

**Results:**

75 patients with PFO-related CS and 147 non-CS patients were enrolled. Multivariate logistic analysis showed that the change in PFO height, large PFO, atrial septal aneurysm (ASA), and sustained right-to-left shunt (RLS) had independent relationships with CS. Based on the odds ratio value of each independent factor, a scoring system was built: change in PFO height ≥ 1.85 mm (3 points), large PFO (2 points), ASA (5 points), sustained RLS (2 points). 0–2 points correspond to low-risk PFO, 3–5 points medium-risk PFO, and 7–12 points high-risk PFO. ROC analysis showed an area under the curve of 0.80 to predict CS. The proportion of patients with CS is increasing based on these points.

**Conclusions:**

Our study screened out the change in PFO height as an independent predictor of CS. A simple and convenient scoring system can provide constructive guidance for identifying whether the PFO is causal and consequently selecting patients more likely to benefit from closure.

## Introduction

It is well known that patent foramen ovale (PFO) is involved in the pathogenesis of many diseases including cryptogenic stroke (CS), migraine, decompression sickness, and arterial deoxygenation syndromes. Randomized controlled trials (RCTs) and long-term follow-up analyses have demonstrated that PFO occlusion could reduce stroke recurrence compared to drug therapy alone (1–4). However, it is well established that there is a high incidence of PFO in common people; thus, the channel might be a chance event rather than a causative one, implying that patient selection is vital before the procedure. To assess the causative relationship, researchers constructed the Risk of Paradoxical Embolism (RoPE) score calculator based on clinical factors, including younger age, no hypertension and diabetes, past cerebrovascular accidents, and smoking. Obviously, it relies deeply on clinical history, ignoring the structural features of the PFO (5). Cerebral ischemic events tend to be caused by larger and longer PFO, but other studies have failed to reach this conclusion (6–11). Although PFO morphology appears to be closely related to disease, studies based on PFO morphology are scarce and morphology-based scoring systems are mixed. The aim of this study was to find risk factors related to CS by comparing the anatomical features of PFO patients in CS and those without CS (non-CS) using transesophageal echocardiography (TEE). In addition, this study developed a scoring system to identify high-stroke risk PFO channels based on echocardiographic features.

## Methods

### Participants

The case group was derived from a prospective multicenter registered clinical trial (http://www.chictr.org.cn/; ChiCTR-ONRC-13003436). This study was approved by the Ethics Committee of the First Affiliated Hospital of Xi'an Jiaotong University, and all patients provided signed informed consent. Between September 2018 and September 2021, a total of 75 patients admitted to our department for PFO-related CS were included. CS was defined as an episode without a major cause, including atherosclerotic stenosis of the large arteries, small artery (lacunar) disease, or cardioembolism (12). PFO-related CS was identified by our neurologist, including evaluation of magnetic resonance imaging, computed tomography, carotid Doppler ultrasonography, and 24-h Holter monitoring, to ensure that PFO is the most plausible stroke pathogenesis. The imaging findings of PFO-related CS include single cortical or multiple small ischemic lesions (<15 mm) in the vertebrobasilar circulation without any visible vascular occlusion on angiography. We excluded patients with large cortico-subcortical infarcts or confluent lesions (>15 mm) with additional lesions in the multi-circulation area (13). Patients with other types of heart defects, thrombophilia, vasculitis, migraine, or cancer-related stroke were also excluded. The control group consisted of 122 consecutive patients with incidental PFO discovered by TEE during the program. Clinical data of the case group were obtained by reviewing the medical information recorded in the structured questionnaire, including age, sex, history of hypertension, diabetes, atrial fibrillation, coronary heart disease, and hyperlipidemia. The control group data were sourced from the medical records of our hospital.

### Echocardiographic studies

Transthoracic echocardiography (TTE), contrast transthoracic echocardiography (cTTE), and TEE were performed by an experienced echocardiographer, and the results were assessed by two investigators who were unaware of whether the patient had a stroke. We utilized TTE to measure the dimensions of the left atrium and aorta. Right-to-left shunt (RLS) grading was defined by microbubbles in the left heart at rest: negative was considered if no microbubbles were observed and sustained shunt if any microbubbles were present. We evaluated the length and height of the PFO channel both at rest and during the Valsalva maneuver, the presence of atrial septal aneurysm (ASA), and hypermobile interatrial septum by TEE, GE-Vivid-E9. The PFO channel length was defined as the maximal visible overlap between the septum primum and secundum. The height of the PFO was measured as the maximum separation between the septum primum and secundum on the left atrial side. Changes in length and height were defined as the value difference between rest and the Valsalva maneuver. The percentage change in length and height were defined as the change in length and height/value at rest ^*^100% (14). ASA was defined as a deviation of >10 mm from the atrial septal plane to the atria or a total deviation of >15 mm. The hypermobile interatrial septum was defined as a moving and floppy septum with a septal offset ≥6.5 mm (15). Large PFO was defined as a height ≥2 mm and long-tunnel PFO was defined as a length ≥ 8 mm (3, 16).

### Statistical analyses

Statistical analyses were conducted using SPSS25.0 (SPSS, IBM Corporation). We presented continuous variables as mean ± standard deviation or median (quartile), and categorical variables as numbers and proportions. We compared continuous variables using the *t*-test or Mann-Whitney *U*-test, and categorical variables using the chi-squared test or Fisher's exact test. The independent risk factors were screened using univariate and multivariate logistic regression analyses. Receiver operating characteristic (ROC) curves were used to transform continuous variables into dichotomous variables using cut-off values that depended on the Youden index and to assess the predictive power of the scoring system.

## Results

### Clinical and echocardiographic data

A total of 222 patients were enrolled, including 75 with PFO-related CS and 147 non-CS. Baseline characteristics were showed in Table 1. The CS group was older and had a higher RoPE score (*P* < 0.001). The two groups were compared based on sex, history of hypertension, diabetes, atrial fibrillation, coronary heart disease, and hyperlipidemia. Table 2 shows echocardiographic information of PFO. The CS group had a larger left atrial dimension and aortic diameter (*P* < 0.05). The PFO channel height in the CS group during the Valsalva maneuver was significantly greater [2.9 (1.9–4.1) vs. 2.0 (1.4–2.8), *P* < 0.001]. The same trends were shown in height change [1.9 (1.1–3.0) vs. 1.0 (0.6–1.5), *P* < 0.001] and in percentage change of height [120 (71–200) vs. 90 (52–150), *P* < 0.05]. The PFO channel length at rest and during the Valsalva maneuver was significantly greater in the CS group [at rest: 8.2 (6.6–11.0) vs. 7.7 (5.6–10.1), *P* < 0.05, during the Valsalva: 9.1 (6.4–11.3) vs. 7.9 (5.5–10.0), *P* < 0.05]. Large PFO, long-tunnel PFO, ASA, hypermobile interatrial septum, and sustained RLS were more prevalent in the CS group (77.3 vs. 44.2%, *P* < 0.001; 62.7 vs. 48.3%, *p* = 0.043; 21.3 vs. 5.4%, *P* < 0.001; 18.7 vs. 8.2%, *P* < 0.021; 53.3 vs. 30.6%, *P* < 0.001, respectively).

**Table 1:** Clinical characteristics of patients.

	**CS**	**non-CS**	***P*** **-value**
	**(*n =* 75)**	**(*n =* 147)**	
Age, years	52 (43~56)	42 (32~53)	< 0.001
Male sex	40 (53.3%)	68 (46.3%)	0.319
Hypertension	21 (28.0%)	27 (18.4%)	0.099
Diabetes mellitus	3 (4.0%)	4 (2.7%)	0.606
Dyslipidemia	2 (2.7%)	10 (6.8%)	0.197
Atrial fibrillation	0 (0.0%)	5 (3.4%)	0.106
Coronary heart disease	5 (6.7%)	3 (2.0%)	0.080
RoPE score	7 (6~8)	6 (4~7)	< 0.001

CS, cryptogenic stroke; RoPE, Risk of Paradoxical Embolism.

**Table 2:** Echocardiographic PFO characteristics.

	**CS**	**non-CS**	***P*** **-value**
	**(*n =* 75)**	**(*n =* 147)**	
Left atrium	27 (25~28)	26 (24~29)	0.021
Aortic sinus	30 (28~32)	29 (26~31)	0.005
Ascending aorta	30 (28~32)	29 (26~31)	0.003
Height at rest	1.1 (0.8~1.7)	1.0 (0.7~1.6)	0.185
Height during the Valsalva	2.9 (1.9~4.1)	2.0 (1.4~2.8)	< 0.001
Height change	1.9 (1.1~3.0)	1.0 (0.6~1.5)	< 0.001
Percentage change of height	120 (71~200)	90 (52~150)	0.004
Length at rest	8.2 (6.6~11.0)	7.7 (5.6~10.1)	0.040
Length during the Valsalva	9.1 (6.4~11.3)	7.9 (5.5~10.0)	0.011
Length change	1.0 (0.4~1.8)	0.7 (0.4~1.4)	0.198
Percentage change of length	11 (4~21)	11 (4~20)	0.664
Large PFO	58 (77.3%)	65 (44.2%)	< 0.001
Long-tunnel PFO	47 (62.7%)	71 (48.3%)	0.043
ASA	16 (21.3%)	8 (5.4%)	< 0.001
Hypermobile interatrial septum	14 (18.7%)	12 (8.2%)	0.021
Sustained RLS at rest	43 (53.3%)	45 (30.6%)	< 0.001

CS, cryptogenic stroke; PFO, patent foramen ovale; ASA, atrial septal aneurysm; RLS, right to left shunt.

### Factors related to CS

Univariate logistic analysis revealed that the dimensions of the aortic sinus and ascending aorta, PFO height during the Valsalva maneuver, change in PFO height, the percentage change in PFO height, PFO length during the Valsalva maneuver, ASA, hypermobile interatrial septum, and sustained RLS were associated with CS. Multivariate logistic analysis showed that changes in PFO height, ASA, and sustained RLS had an independent relationship with CS (Table 3).

**Table 3:** Univariate and multivariable regression logistic analyses for selection of echocardiographic PFO characteristics associated with stroke.

	**Univariate analysis**		**Multivariable analysis**	
	**OR (95%CI)**	***p*** **-value**	**OR (95%CI)**	***p*** **-value**
Left atrium	1.10 (1.0–1.20)	0.055	1.05 (0.93–1.18)	0.448
Aortic sinus	1.13 (1.04–1.23)	0.004	1.08 (0.93–1.26)	0.316
Ascending aorta	1.12 (1.04–1.21)	0.003	0.98 (0.85–1.14)	0.809
Height during the Valsalva	1.52 (1.23–1.88)	< 0.001	0.80 (0.54–1.19)	0.267
Height change	2.24 (1.04–4.82)	0.038	2.36 (1.30–4.28)	0.005
Percentage change of height	1.74 (1.27–2.38)	0.001	1.02 (0.64–1.61)	0.939
Length at rest	1.08 (1.0–1.18)	0.063	0.97 (0.77–1.21)	0.778
Length during the Valsalva	1.12 (1.03–1.23)	0.011	1.11 (0.87–1.41)	0.418
Hypermobile interatrial septum	2.58 (1.13–5.91)	0.025	1.79 (0.67–4.76)	0.245
ASA	4.71 (1.91–11.61)	0.001	4.60 (1.65–12.82)	0.003
Sustained RLS	3.05 (1.71–5.42)	< 0.001	2.33 (1.20–4.52)	0.013

PFO, patent foramen ovale; ASA, atrial septal aneurysm; RLS, right to left shunt.

We defined 1.85 mm of change in PFO height as the optimal cut-off value with an area under the curve (AUC) of 0.73 to predict PFO-related CS (see Supplementary Figure 1). When dichotomous parameters were added to the multivariate logistic analysis, changes in PFO height, ASA, sustained shunts at rest, and large PFO were independently associated with CS. In addition, the same echocardiographic features were independently associated with CS when age was added as a variable in multivariate logistic analysis (Table 4).

**Table 4:** Multivariable regression logistic analyses based on dichotomous parameters of PFO characteristics.

	**Multivariable analysis 1**		**Multivariable analysis 2**	
	**OR (95%CI)**	***p*** **-value**	**OR (95%CI)**	***p*** **-value**
Left atrium	1.08 (0.96–1.22)	0.210	1.09 (0.96–1.23)	0.198
Aortic sinus	1.07 (0.91–1.25)	0.406	1.07 (0.92–1.26)	0.368
Ascending aorta	0.98 (0.85–1.14)	0.828	0.95 (0.81–1.12)	0.566
Height during the Valsalva	1.22 (0.83–1.79)	0.311	1.22 (0.83–1.79)	0.312
Large PFO	2.24 (1.04–4.82)	0.038	2.28 (1.06–4.91)	0.036
Long-tunnel PFO	1.18 (0.61–2.30)	0.625	1.19 (0.61–2.31)	0.617
Hypermobile interatrial septum	1.53 (0.56–4.15)	0.405	1.44 (0.53–3.90)	0.475
ASA	5.01 (1.72–14.58)	0.003	4.68 (1.60–13.71)	0.005
Sustained RLS	2.14 (1.11–4.16)	0.024	2.09 (1.08–4.07)	0.030
Height change≥1.85mm	2.76 (1.17–6.47)	0.020	2.57 (1.08–6.12)	0.033
Age			1.02 (0.99–1.05)	0.320

PFO, patent foramen ovale; ASA, atrial septal aneurysm; RLS, right to left shunt.

### PFO scoring system

**Figure 1:**
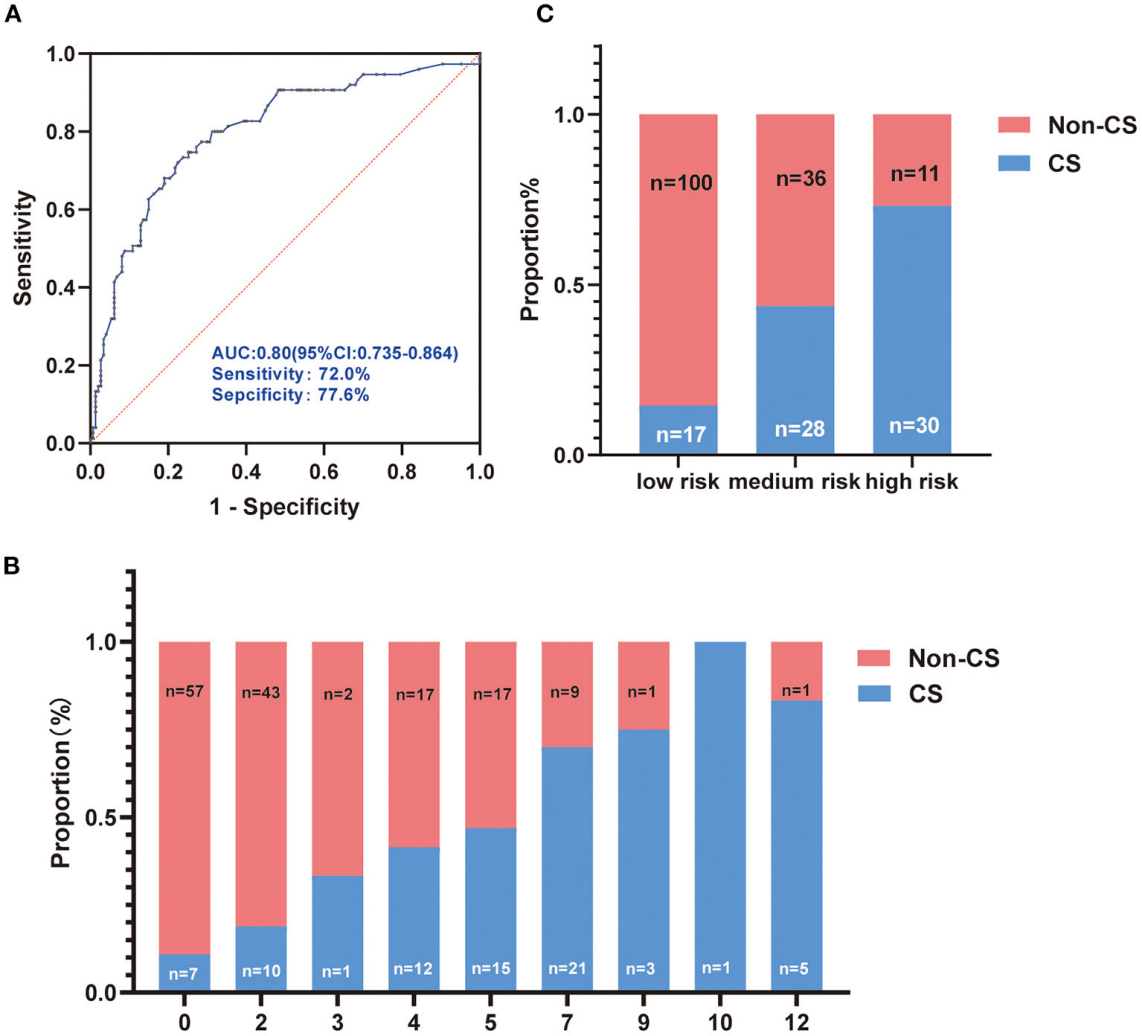
Evaluation of the score system. **(A)** Receiver operating characteristic of the score system for predicting cryptogenic stroke (CS); **(B)** The proportion of CS and no-CS patients according to the score system; **(C)** The score system was divided into low, medium, and high-risk grades based on the proportion of 0–25%, 25–50%, and 50–100%.

We built a scoring system based on the odds ratio (OR) value of each independent factor related to the CS as shown in Table 5. Subsequently, we estimate the scoring system. ROC analysis presented an AUC of 0.80 (95% confidence interval = 0.735–0.864) to predict CS (Figure 1A). The proportion of CS patients increased with this point, as shown in Figure 1B. Based on the average probabilities of 0–25%, 25–50%, and 50–100% (14), we divided the system into low-, medium-, and high-risk grades, which matched the score point ranges: 0–2, 3–5, and 7–12, respectively (Figure 1C).

**Table 5:** A calculator for CS prediction based on OR value.

**Variables**	**Points**
Large PFO	2
ASA	5
Sustained RLS	2
Height change ≥ 1.85 mm	3

PFO, patent foramen ovale; ASA, atrial septal aneurysm; RLS, right to left shunt.

## Discussion

Based on assessing the characteristics of PFO in patients with CS and non-CS, we obtained the following findings: (1) change in PFO height, large PFO, ASA, and sustained RLS had independent relationships with CS; (2) a scoring system was proposed to evaluate the extent of the pathogenic relationship between PFO and CS. This study emphasized the understanding that PFO anatomical features are associated with CS, and the scoring system could help identify optimal patients who can benefit from PFO occlusion.

TEE, a semi-invasive examination, is considered the gold standard for diagnosing PFO. Clinical settings and scientific research have focused on the height and length of the PFO at rest and after Valsalva maneuvers under TEE, however, the situation between these two states is rarely studied. A larger PFO channel diameter promotes more RLS, along with greater risk and severity of stroke (8, 17, 18). Based on the initial belief that a PFO opening diameter ≥ 4 mm has relationship with stroke, more than 2 mm is now used as the dividing line in studies (3, 6). Studies have shown that closure therapy with a PFO diameter ≥ 2 mm can reduce the stroke recurrence rate (3). Our study proved that a PFO height ≥ 2 mm was an independent risk factor for CS. The large PFO channel allows more and larger emboli to travel through the interatrial septum until the peripheral system following RLS, which is the most common mechanism of cerebral infarction. Because each individual's atrial pressure, the activity of the primary septum, and the combined anatomical characteristics of the atrial septum are different, all of the above will lead to different PFO openings after the Valsalva maneuver, as well as variations in the change in PFO channel height. A larger change in PFO height between the two states could induce more RLS, implying a greater likelihood of paradoxical embolism over a certain period. This might explain why a change in height acts as an independent risk factor. This is the first study to find an association between the diameter change of PFO and CS, prompting researchers to focus on the dynamic changes in the channel.

The presence of ASA was more common in CS patients (19), and those combined with PFO and ASA presented with a higher OR for stroke and severity of clinical status (20, 21). Meta-analysis of RCTs found the benefit of the PFO closure was significantly greater in patients with ASA (RR 0.17 [95% CI 0.06–0.53]), and substantial RLS (RR 0.23 [95%CI 0.11–0.48]) (22). The amount of shunt mainly reflects the size of the PFO (23). Since Valsalva maneuvers are largely dependent on the standard degree of patient manipulation and their heterogeneity may affect the results of morphological characteristics of the PFO, we tested the grading of microbubbles at rest to circumvent this deficit. Univariate and multivariate logistic analyses confirmed that sustained shunting is an independent risk factor for CS. A recently published observational study showed that ASA had greater importance than shunt size when predicting recurrent stroke (24). Our results support this finding, showing a higher OR for ASA. In addition, Beyls et al. (25) found that the width of the aorta may be a risk factor for stroke by affecting atrial septal mobility, and our multivariate analysis found it to be a risk factor but not an independent factor.

Based on the selected factors, we built a scoring system with a satisfying AUC to predict PFO-related CS, and it also had excellent internal validation ability. Several scoring models have been proposed in the literature. The Risk of Paradoxical Embolism (RoPE) score was the first model built for the purpose of selecting optimal patients who could benefit from occlusion therapy; however, RoPE is based on the medical history and neuroimaging findings, ignoring the morphological characteristics of PFO (5). To reduce reliance on cranial imaging, the clinical RoPE (cRoPE) score was proposed with a performance comparable to that of RoPE (26), but the latter deficiency remained. Nakayama et al. (11) retrospectively evaluated the PFO characteristics in 57 patients with CS using TEE to build a high-risk PFO scoring system. They selected long-tunnel PFO, hypermobile interatrial septum, prominent Eustachian valve or Chiari's network, large RLS during the Valsalva maneuver, and low-angle PFO as the independent factors. After assigning each factor 1 point, a score ≥2 points was distinguished as high-risk PFO with CS. However, they did not incorporate the PFO dynamic change indicator, and a simple method of assigning score points to selected factors might have missed the contribution of each indicator. The Morphological Stroke Factors of PFO (MorPFO) score was newly constructed depending on PFO channel length reduction, short septum secundum, thin septum primum, large RLS, low PFO channel length/height ratio, and the presence of an ASA (14). Although it has pioneered the use of indicators that reflect the dynamic changes in PFO, the predictors were relatively complex and difficult to calculate, making clinical promotion challenging. Based on TEE, we screened out three commonly used indicators and one previously underappreciated indicator to establish a scoring system that is simple to calculate and convenient for clinical use. Despite advances in PFO research, there are still many unanswered questions, such as the selection of optimal patients for closure who at the highest risk of stroke (27). The system might help to identify whether the PFO is causal and consequently select patients more likely to benefit from closure. In addition to the anatomical morphology of the PFO, other stroke risk factors and the structures surrounding the PFO which might interfere with the result, such as age, cardiovascular history, the left atrium, and aorta dimensions, were considered.

The study still had limitations. Although this was a prospective study, it had a relatively small sample size, which may have negatively affected the results. We missed external validation of the score which will schedule in the future. To avoid errors induced by the number of counts on cTTE, our study adopted a more direct classification method, positive or negative, ignoring the effect of different degrees of shunting on the results. Additionally, all TEE was performed without sedation, which may have affected the predictive power of the scoring system in the anesthesia population.

## Conclusion

Changes in PFO height, large PFO, ASA, and sustained RLS were independently related to CS. The scoring system based on the four independent anatomical factors built here can be used to confirm the pathogenicity of PFO and is beneficial to screen patients for occlusion.

## Data availability statement

The original contributions presented in the study are included in the article/Supplementary material, further inquiries can be directed to the corresponding author.

## Ethics statement

The studies involving human participants were reviewed and approved by Ethics Committee of the First Affiliated Hospital of Xi'an Jiaotong University. The patients/participants provided their written informed consent to participate in this study.

## Author contributions

XL and YusZ were the principal investigators, conducted the statistical analysis, and drafted the manuscript. JS, LS, BL, and XX performed data collection. XL, HX, HZ, and YusZ edited and revised the manuscript. All authors approved the submitted version.

## Conflict of interest

The authors declare that the research was conducted in the absence of any commercial or financial relationships that could be construed as a potential conflict of interest.

## Publisher's note

All claims expressed in this article are solely those of the authors and do not necessarily represent those of their affiliated organizations, or those of the publisher, the editors and the reviewers. Any product that may be evaluated in this article, or claim that may be made by its manufacturer, is not guaranteed or endorsed by the publisher.
