# Supplementary figures and images for: Change in patent foramen ovale height is associated with cryptogenic stroke and the construction of a morphology-based scoring system
Source: Front Cardiovasc Med. 2022 Nov 28;9:1010947. doi: 10.3389/fcvm.2022.1010947 (PMC9742367; doi:10.3389/fcvm.2022.1010947)

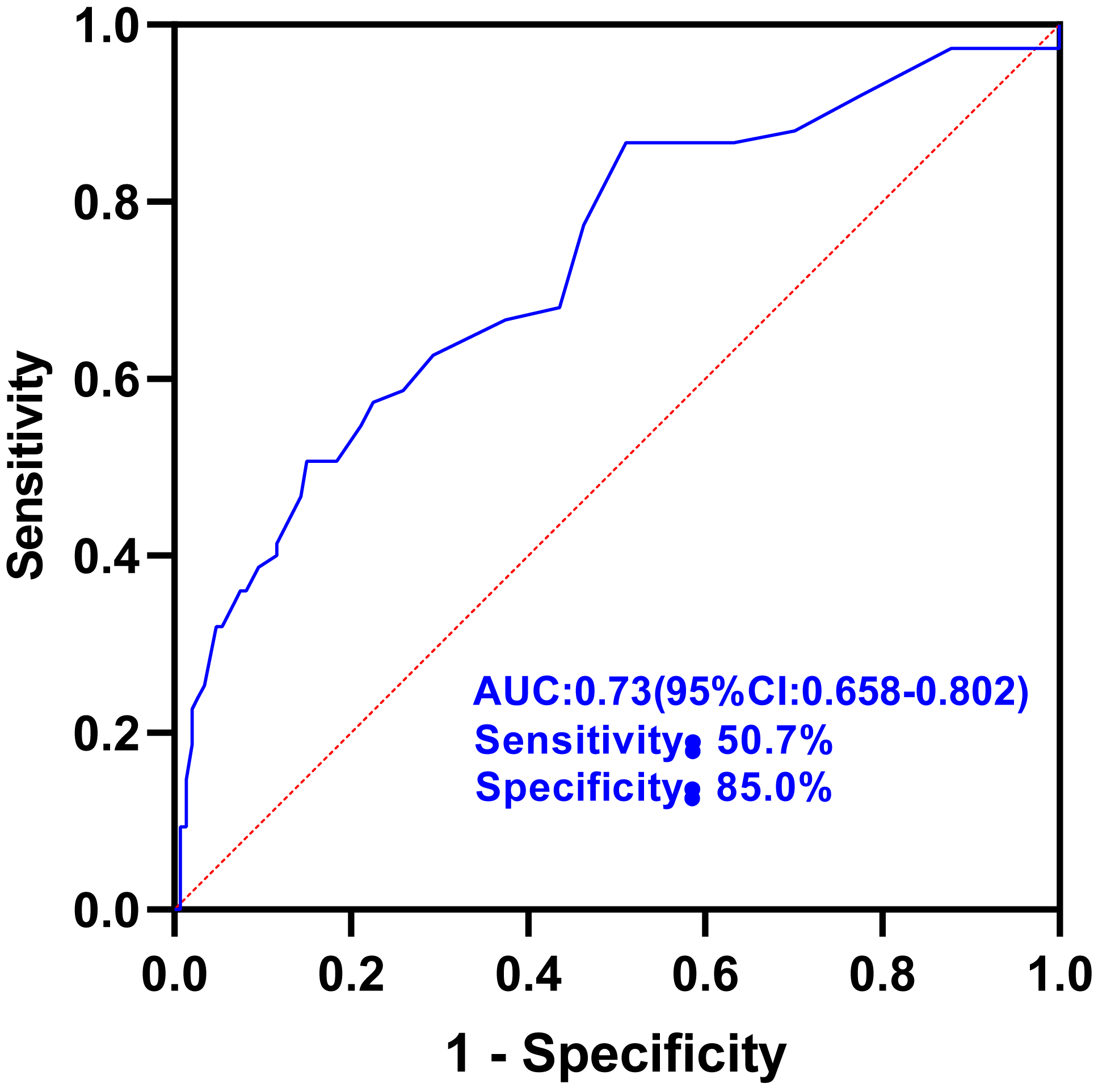

Supplement: Supplementary file 1 [file Image_1.TIF]
